# A study on the prevalence of type 2 diabetes in coastal Karnataka

**DOI:** 10.4103/0973-3930.62597

**Published:** 2010

**Authors:** Chythra R. Rao, Veena G. Kamath, Avinash Shetty, Asha Kamath

**Affiliations:** Department of Community Medicine, Bio-statistics, Kasturba Medical College, Manipal - 576 104, Karnataka, India

**Keywords:** Prevalence, socio-demographic correlates, type 2 diabetes

## Abstract

**Aim:**

To estimate the prevalence and study the socio-demographic correlates of type 2 diabetes among adults aged 30 years and above.

**Setting and Design:**

A cross-sectional community-based survey, among individuals of either sex, aged 30 years and above was carried out in the field practice area of a medical college.

**Methods and Materials:**

The study was carried out on 1,239 respondents, using a two-stage, stratified, random sampling technique. Data was collected by a personal, face-to-face interview followed by blood sugar estimation using a glucometer.

**Statistical Analysis:**

Was performed by using the Statistical Package for Social Sciences (SPSS) version 11.5.

**Results:**

The overall prevalence of diabetes was 16%. Self-reported diabetes was 11.2%, while 4.8% of previously normal people were found to have high fasting capillary blood glucose levels. Increasing age showed two-fold, four-fold, and six-fold higher odds for 40 – 49, 50 – 59, and ≥ 60 years age group, respectively, as compared to the 30 - 39 year age group (*P* < 0.001). Nineteen percent of the males had diabetes, (OR = 1.38, 95% CI = 1.01 – 1.88). In the high socioeconomic strata, 32% of the subjects had diabetes (*P* = 0.018 unadjusted odds ratio 3.29, 95% CI = 1.40 – 7.74).

**Conclusion:**

The high prevalence of diabetes in this coastal population needs further evaluation.

## Introduction

Diabetes mellitus (DM) has reached epidemic proportions globally.[1] The World Health Organization (WHO) estimated that there were 135 million diabetic individuals in the year 1995 and it has been projected that this number will increase to 300 million by the year 2025.[2] WHO has projected that the maximum increase in the number of diabetics would occur in India. Considering the large population and increasing prevalence of diabetes mellitus of nearly 33 million diabetic subjects, the burden of diabetes in India could be enormous.[3] With a high genetic predisposition and high susceptibility to environmental insults, the Indian population faces a higher risk of diabetes and its associated complications.[4] In the absence of an efficient non-communicable disease (NCD) surveillance system in our country, the only reliable method of obtaining disease estimates is to conduct field studies. Epidemiological studies are urgently needed in each region of India to have a baseline against which future trends in risk-factor levels can be assessed, and preventive strategies planned. There is paucity of such data in the state and the literature review does not reveal many such studies from our area. In view of addressing the demand for this need, the present study was undertaken, with the objective of determining the prevalence of diabetes and to study the association between the various correlates for diabetes.

## Materials and Methods

We conducted a cross-sectional, community-based survey over a period of 15 months (from August 2006 – October 2007), among individuals of either sex, aged 30 years and above. The study was carried out in the field practice area of a medical college in Karnataka. The field practice area covered a population of 45,587 living in 7,164 families spread out in 11 villages. It was situated along the coastal area in the southern part of India. The population in these villages was homogeneous in terms of occupation, socioeconomic status, and food habits and the findings of one village could be extrapolated to all the other villages.

The study population included all men and women aged 30 years and above. Individuals less than 30 years were not included, due to the low prevalence of type 2 diabetes in this age group.[5,6] Study variables included socio-demographic characteristics, physical activity, blood pressure, anthropometric measurements, family history of diabetes, and blood glucose measurements. Pregnant or lactating women up to 12 weeks post-partum were excluded from the study, due to the possible impaired glucose tolerance status in this group, due to pregnancy.

Considering a prevalence of 3.8% for diabetes in rural adults, with an allowable error of 30%, and 95% confidence level, the estimated sample size was 1,081. A non-response rate of 20% required a sample of 1,351 to be studied. A two-stage, stratified probability, proportional to the size sampling technique was used to select the study sample. In the first stage, two villages were selected from the field practice area based on the investigator's convenience. In the second stage, random samples of the study subjects were drawn from each locality of the selected village, proportional to their population sizes. Institutional ethical committee clearance was obtained prior to the initiation of the study. The identification of the localities and households was done with the help of a field auxiliary nurse midwife (ANM). The selection of the lane and first house, within the locality was done by random selection, by employing the procedure described in the cluster sampling technique used for evaluation of universal immunization coverage.[7] Starting from the first house onwards all the houses within the lane were covered continuously, keeping toward the left. This procedure was continued until the sample size for each locality was obtained. Written informed consent was obtained from all the subjects. During house visits, the objectives of the study were explained to the eligible household members and data was collected by personal, face-to-face interviews, using a pre-designed questionnaire. The questionnaire included details on socio-demographic variables, past / family history of diabetes, and physical activity status.[8] The socioeconomic status was assessed using the modified Uday-Parikh scale.

Weight was recorded using a standard weighing scale (Krups weighing scale, New Delhi, India) that was kept on a firm horizontal surface. Weight was recorded to the nearest 500 gm. Height was recorded using a measuring tape to the nearest 1 cm. Subjects were requested to stand upright without shoes with their back against the wall, heels together and looking forward. Body mass index (BMI) was calculated using the formula, weight (kg) / height (m^2^). Waist circumference was measured to the nearest 0.1 cm at the mid-point between the costal margin and iliac crest using a non-stretchable measuring tape, at the end of normal expiration with the subject standing erect in a relaxed position, feet 25 – 30 cm apart. Hip circumference was measured at the level of the greater trochanters (widest portion of the hip) to the nearest 0.1 cm with a measuring tape, while the subject was standing with the arms by the side and feet together. Waist-hip ratio was calculated as the ratio of waist circumference over hip circumference.[9]

A person was considered to be obese if body mass index (BMI) ≥ 25 kg/m^2^ and overweight when BMI ≥ 23 kg/m^2^. Central / abdominal obesity was considered to be present when waist circumference ≥ 90 cm in males and ≥ 80 cm in females. Waist hip ratio of > 1.0 for males and > 0.85 for females was defined as truncal obesity.[10,11]

Blood pressure was measured on the right arm in a sitting posture, with the subject in a relaxed state. Standardized mercury sphygmomanometer (Diamond deluxe BP apparatus, Pune, India) with adult size cuff was used. The first appearance of (phase 1 of korotkoff sounds) sound was used to define Systolic Blood Pressure (SBP). The disappearance of sound (phase 5) was used to define Diastolic Blood Pressure (DBP). Two readings were taken five minutes apart and the average of the two readings was taken as the final blood pressure reading. A person was considered to be a hypertensive if he / she was an already diagnosed case of hypertension and / or on treatment or with a current SBP of ≥ 140 mm Hg or DBP ≥ 90 mm Hg (JNC VII criteria).[12]

Random blood glucose (RBG) for the subjects was estimated at the time of the interview by using a standardized digital glucometer (Accu Chek, Roche diagnostics, Germany), using the capillary finger prick method. Subsequently, on a pre-informed date, fasting blood glucose (FBG) estimation was done for those subjects in whom RBG ≥ 110 mg/dl was obtained. RBG estimation was done even for people with diabetes, irrespective of their RBG values, they were not tested further. A person was considered to be having diabetes if he / she was an already diagnosed case of diabetes and / or on treatment or current fasting capillary blood glucose ≥ 110 mg/dl. (Fasting being defined as no caloric intake for at least eight hours).[13,14] Blood pressure and blood glucose estimation was done for the individuals of the household irrespective of whether they had diabetes / hypertension.

Individuals with either a parent or a sibling (brother or sister) having diabetes, were considered to have a positive family history. Eligible subjects unavailable during the first house visit were approached on another pre-informed date as per their convenience. Even after two such visits if the subject was non-compliant, then he / she was considered as a non-respondent.

### Statistical Methods

Prevalence of diabetes and risk factors of diabetes are presented as percentages. A Chi-square test for trend was used to assess the trends in the prevalence of diabetes among different age groups, while a chi-square test was used to study the association of prevalence of diabetes and the different correlates. To study the impact of the selected socio-demographic factors, anthropometric measurements (BMI) and other risk factors, on the prevalence of diabetes, we performed multiple logistic regression analysis, with diabetes as a dichotomous outcome, and age, sex, religion, occupation, socioeconomic status, physical activity, positive family history of diabetes, history of current hypertension, BMI, and central obesity as independent variables. All statistical analysis were performed using the Statistical Package for Social Sciences (SPSS) version 11.5. A *P*-value < 0.05 was considered significant.

## Results

The baseline characteristics of the study subjects are as shown in Table 1. The study included 1,419 subjects with a response rate of 87.3%. The total sample studied was 1,239, of which 434 (35%) were males and 805 (65%) females. There was inadequate representation of males in the study sample as most of them were employed overseas or in the neighboring states and many were involved in occupations such as fishing and unskilled daily wage labor and thus, were not available during the survey. Of the total study subjects, 85.6% were Hindus, 8.6% Muslims, and 5.7% Christians. The literate proportion in the sample was 81.2%, out of whom 75.9% were females and 91% were males. The socioeconomic status assessed by the modified Uday-Parikh scale for rural areas showed that 70.1% belonged to the middle class, 27.6% to the lower class, and 2.3% to the upper class. A sedentary lifestyle was observed in 11.1% of the subjects, while 41.8% were engaged in moderate physical activity. Positive family history of diabetes was present in 26% of the individuals. Over half (57.3%) of the study population had a normal BMI, while the overweight category included 14.6% of the subjects. In the study, 28.1% of the individuals were found to be obese when BMI was used as the defining criteria, but over half of the subjects had abdominal and truncal obesity (56.2 and 62.1%, respectively).

**Table 1:** Characteristics of the study subjects

Variables	Males No. (%) N = 434	Females No. (%) N = 805	Total No. (%) N = 1239
Age group (Yrs.)			
30 – 39	115 (26.5)	252 (31.3)	367 (29.6)
40 – 49	123 (28.4)	183 (22.8)	306 (24.6)
50 – 59	77 (17.7)	138 (17.1)	215 (17.4)
≥ 60	119 (27.4)	232 (28.8)	351 (28.4)
Literacy			
Illiterate	39 (9.0)	194 (24.1)	233 (18.8)
Primary (1 – 4)	68 (15.7)	128 (15.9)	196 (15.8)
Secondary (5 – 12)	282 (65.0)	452 (56.1)	734 (59.3)
Graduation and above	45 (10.3)	31 (3.9)	76 (6.1)
Occupation			
Unskilled	82 (18.9)	219 (27.3)	301 (24.3)
Unemployed and Retired	117 (27.0)	6 (0.7)	123 (9.9)
Skilled	178 (41.0)	26 (3.3)	204 (16.5)
Service	57 (13.1)	9 (1.1)	66 (5.3)
Housewife	0	545 (67.7)	545 (44.0)
Physical activity			
Sedentary	70 (16.1)	67 (8.3)	137 (11.1)
Light	131 (30.3)	372 (46.3)	503 (40.6)
Moderate	166 (38.2)	352 (43.7)	518 (41.8)
Heavy	67 (15.4)	14 (1.7)	81 (6.5)
Family history of diabetes	117 (27)	205 (25.5)	322 (26.0)
BMI*			
< 22.9	241 (55.5)	467 (58.2)	708 (57.3)
23.0 – 24.9	75 (17.3)	106 (13.2)	181 (14.6)
≥ 25	118 (27.2)	229 (28.6)	347 (28.1)
Waist and hip measurements			
Central obesity	134 (30.9)	562 (69.8)	696 (56.2)
Truncal obesity	58 (13.4)	711 (88.3)	769 (62.1)

* BMI – Body Mass Index (kg/m^2^)

The overall prevalence of diabetes was found to be 16%. Among males, 18.8% were found to have diabetes, as compared to 14.4% among females. Self-reported diabetes was 11.2%, while 4.8% of previously normal people were found to have high fasting capillary blood glucose levels (new cases), based on the screening criteria employed.

Chi-square for trend showed significant association between increasing age and diabetes (χ^2^_trend_-71.21, *P* < 0.001). The Chi-square test used to study the association between the prevalence of diabetes among those with positive family history those with different physical activity status; and those found to be obese were found to be statistically significant (*P* < 0.001).

Results of the multivariate logistic regression model that examined the cross-sectional correlates of diabetes are presented in Table 2. Increasing age, Muslims, people engaged in service jobs or skilled profession, sedentary lifestyle, positive family history of diabetes, history of current hypertension, and being obese, as defined by BMI, and those having central obesity were associated with a high risk of having diabetes.

**Table 2:** Summary table of significant correlates for diabetes

Variablea	Unadjusted OR	95% C.I	Adjusted OR	95% C.I
Age group (yrs)				
30 – 39	1.00	1.00	1.00	1.00
40 – 49	2.67	1.51 – 4.73	2.05	1.11 – 3.76
50 – 59	4.71	2.67 – 8.32	3.76	2.01 – 7.00
≥ 60	6.89	4.10 – 11.57	5.47	2.90 – 10.30
Gender				
Female	1.00	1.00	-	-
Male	1.38	1.01 – 1.88	-	-
Religion				
Hindu	1.00	1.00	1.00	1.00
Muslim	2.47	1.57 – 3.89	2.08	1.23 – 3.50
Christian	1.91	1.07 – 3.38	1.58	0.84 – 2.99
Occupation				
Unskilled	1.00	1.00	1.00	1.00
Unemployed	5.04	2.81 – 9.05	1.31	0.63 – 2.73
Skilled	2.44	1.38 – 4.33	2.22	1.18 – 4.17
Service	4.05	1.99 – 8.26	2.94	1.29 – 6.69
Housewife	2.57	1.58 – 4.19	0.84	0.47 – 1.50
Socioeconomic status				
Low	1.00	1.00	-	-
Middle	1.40	0.97 – 2.02	-	-
High	3.29	1.40 – 7.74	-	-
Physical activity				
Heavy	1.00	1.00	1.00	1.00
Moderate	2.24	0.78 – 6.36	1.50	0.49 – 4.56
Light	4.54	1.62 – 12.71	2.22	0.71 – 6.90
Sedentary	9.10	3.13 – 26.47	3.59	1.07 – 11.99
Positive family history of diabetes	1.85	1.34 – 2.56	1.84	1.26 – 2.68
Currently hypertensive	3.40	2.46 – 4.71	1.66	1.14 – 2.42
BMI				
< 22.9	1.00	1.00	1.00	1.00
23.0 – 24.9	1.88	1.19 – 2.98	1.15	0.68 – 1.95
≥ 25	3.43	2.44 – 4.83	1.88	1.19 – 2.98
Central obesity	3.01	2.12 – 4.29	2.30	1.41 – 3.73

*BMI – Body Mass Index (kg/m^2^)

## Discussion

Epidemiological data from different parts of India show a rising prevalence of diabetes. The clinical diagnosis of diabetes is often prompted by symptoms such as increased thirst and urine volume, recurrent infections, unexplained weight loss and in severe cases drowsiness and coma. For clinical purposes, an Oral Glucose Tolerance Test (OGTT) to establish diagnostic status need only be considered if casual blood glucose values lie in the uncertain range (i.e., between the levels that establish or exclude diabetes) and fasting blood glucose levels are below those which establish the diagnosis of diabetes.[13] Oral glucose tolerance testing (OGTT), although still a valid mechanism for diagnosing DM, is not recommended as part of the routine care, as OGTT may be difficult to perform in field studies and the cost and demands on participants' time may be excessive. Therefore, for epidemiological studies, the revised criteria for the diagnosis of diabetes emphasize fasting plasma glucose (FPG) as a reliable and convenient test for diagnosing DM in asymptomatic individuals.[15]

Our study was planned as a feasibility study, the first of its kind in our taluk, to determine community-based prevalence of the rising problem of diabetes. In a setting of limited resources and socio-demographic characteristics of the local population, FPG was considered as a reliable and convenient test for diagnosing DM in asymptomatic individuals.

Our study findings were in contrast to the findings reported by other Indian studies, with respect to the high prevalence of type 2 diabetes mellitus and the proportion of the known and newly detected cases of diabetes.[16–23] In the present study, the criteria employed for screening (fasting plasma glucose) FPG >126 mg/dl, equivalent to the capillary value of 110 mg/dl, differed from the earlier reported data, which had employed FPG >140 mg/dl. Secondly > 30-year-old individuals were assessed in our study, while the others had mostly included > 20-year-old subjects, thereby, the overall prevalence reported for diabetes would be less, as the prevalence of diabetes in the 20 – 30 year age group is low (1 – 3%).[5,6] An attempt to quantify lifestyle changes contributing to the disease status was not made in the present study. A combination of these factors could be responsible for the glaring difference in the reported prevalence. The recent CURES study, conducted in urban south India has reported a prevalence of 15.5%,[24] which is comparable to our study findings. A study from Saudi Arabia also reported similar findings, but the females with diabetes outnumbered the males.[25]

Male preponderance and age-wise increase in prevalence noted in our study have been reported previously on numerous studies.[5,6,19,22,26]

In this study multivariate logistic regression analysis identified increasing age, Muslims, a skilled or professional job, sedentary lifestyle, positive family history of diabetes, history of current hypertension, and being overweight or obese as significant correlates for diabetes, among the different variables considered to be significant, which was in conformity with previously reported cross-sectional data.[16–20]

Our study was a community-based, cross-sectional study, the first of its kind in our area to the best of our knowledge, as there is no reported data on the prevalence of diabetes. Estimation of blood glucose levels was done by a single trained investigator in order to have a uniform pattern of sample collection. The authors do agree that there was inadequate representation of males in the study sample and that lower precision levels were selected for sample size calculation. An attempt to quantify the lifestyle changes was also not made in the present study. Although we accept these shortcomings, the house-to-house coverage of over a thousand individuals and the collection of early morning fasting blood sample was the best that could be done with the limited resources. There is a need to establish the base-line risk of population, in order to plan for intervention strategies. This study is a stepping stone in that direction.

## Conclusion

A high prevalence of diabetes was noted in this coastal population. It is also true that changes have been noted in the lifestyle of the population, which could have contributed to the above finding. There have not been similar studies in the past in the same region, against which comparisons could be made. Therefore, future research in this direction is the need of the hour.
